# Vancomycin-resistant enterococcus infection in the hematopoietic stem cell transplant recipient: an overview of epidemiology, management, and prevention

**DOI:** 10.12688/f1000research.11831.1

**Published:** 2018-01-02

**Authors:** Esther Benamu, Stanley Deresinski

**Affiliations:** 1 Division of Infectious Diseases, Department of Medicine, University of Colorado, Aurora, USA; 2 Division of Infectious Diseases and Geographic Medicine, Department of Medicine, Stanford University School of Medicine, Stanford, USA

**Keywords:** Vancomycin resistant enterococcus, Hematopoietic stem cell transplantation, bacteremia

## Abstract

Vancomycin-resistant *enterococcus* (VRE) is now one of the leading causes of nosocomial infections in the United States. Hematopoietic stem cell transplantation (HSCT) recipients are at increased risk of VRE colonization and infection. VRE has emerged as a major cause of bacteremia in this population, raising important clinical questions regarding the role and impact of VRE colonization and infection in HSCT outcomes as well as the optimal means of prevention and treatment. We review here the published literature and scientific advances addressing these thorny issues and provide a rational framework for their approach.

## Introduction

Resistance to vancomycin in *enterococci* was first identified in isolates recovered in 1986, three decades after the introduction of this glycopeptide antibiotic ^1^. Since then, there has been a progressive, albeit geographically heterogeneous, increase in the prevalence of resistance, with among the highest rates in the world seen in the US, where vancomycin-resistant *enterococci* (VRE) is now one of the leading causes of nosocomial infections. VRE represent approximately one-third of *Enterococcus* isolates ^2, 3^, causing an estimated 1,300 deaths each year ^4^. Gastrointestinal (GI) colonization is frequent, and VRE bacteremia (VREB) has become a clinically significant complication in patients undergoing hematopoietic stem cell transplantation (HSCT). Chemotherapy-induced mucositis, neutropenia, prolonged and repeated hospitalizations, antibiotic exposure for therapeutic and prophylactic purposes (particularly with prophylactic antimicrobials with limited activity against Gram-positive [GP] organisms), and the widespread use of central venous catheters are some of the factors that place HSCT recipients at risk for VRE colonization and infection. In the last decade, numerous changes have occurred in the prevention and management of VRE infection, including the development of screening strategies together with attempts at decolonization and the advent of new antibiotics with activity against this organism. At the same time, the introduction of cord blood grafts and non-myeloablative conditioning regimens has resulted in an expansion of the pool of HSCT candidates, now also including older patients.

The emergence of VRE as a major cause of bacteremia in HSCT recipients has raised important clinical questions regarding the optimal means of prevention, the role of VRE colonization in predicting bacteremia, treatment, and the impact on HSCT outcomes.

We review the published literature addressing these aspects and summarize the latest advances in the prevention and treatment of invasive VRE infection in the HSCT recipient.

## Vancomycin-resistant enterococci colonization and infection in hematopoietic stem cell transplant recipients

### Incidence, mortality, and common presentations of vancomycin-resistant enterococci infection

Reported rates of VREB in HSCT recipients have ranged from 1.4–25% ^5– 11^, with more recent studies reporting prevalence rates of 10–15% ( Table 1). VRE has become the leading cause of bloodstream infection (BSI) among allogeneic HSCT recipients, especially in the early post-transplant period ^7, 12, 13^. At the Memorial Sloan Kettering Cancer Center (MSKCC) in New York, VRE was the most frequent cause of bacteremia in the first 35 days post-transplant by a threefold margin during the period of 2004–2006 and represented 53% of early BSIs in 2008–2009 ^7, 12^.

**Table 1.** Vancomycin-resistant enterococci in hematopoietic stem cell transplant recipients: colonization, bacteremia, risk factors, and outcomes. ALL, acute lymphocytic leukemia; allo-HSCT, allogeneic hematopoietic stem cell transplant; AML, acute myeloid leukemia; auto-HSCT, autologous hematopoietic stem cell transplant; BJH, Barnes Jewish Hospital; BSI, bloodstream infection; Cdiff, *Clostridium difficile*; CML, chronic myeloid leukemia; CMV, cytomegalovirus; Colo, colonization; CS, comorbidity score; GC, glucocorticoid; GVHD, graft-versus-host disease; HSCT, hematopoietic stem cell transplant; HR, hazard ratio; IS, immunosuppression; LOS, length of hospital stay; MAR, myeloablative regimen; MDACC, Monroe Dunaway Anderson Cancer Center; MDS, myelodysplastic syndrome; Mort (Colo), mortality in colonized patients; Mort (Colo to BSI), mortality in patients progressing from colonization to bloodstream infection; Mort (non-Colo), mortality in non-colonized patients; MSKCC, Memorial Sloan Kettering Cancer Center; OR, odds ratio; PBSC, peripheral blood stem cell; PCR, polymerase chain reaction; RF, risk factor; RR, relative risk; TBI, total body irradiation; UCB, umbilical cord blood; UConn, University of Connecticut Health Center; URD, unrelated donor; VRE, vancomycin-resistant enterococci; VSE, vancomycin sensitive enterococci.

Study	Sample size, type of HSCT, years of study	Prophylactic antibiotics?	VRE Screening	Colonization rate	Risk factors	VRE BSI incidence	Progression to VRE BSI	Risk factors for VRE BSI	VRE BSI outcomes: mortality (Mort), attributable mortality (Att Mort), and overall survival (OS)
Kapur *et al*. ^16^ 2000 UConn	321 Auto-HSCT 1993–1998	Yes	Rectal swab or stool culture weekly	50% (15/29)	-	3%	27%	-	Mort: 70% Att Mort: 10%
Almyroudis *et al*. ^11^ 2005 MSKCC	298 (adult and children) HSCT 1999–2003	No	-	-	-	Pre-engraftment: 22% (25.7% in adults) Post-engraftment: 19.5%	-	Pre-engraftment: CML (OR=4.13, *P* = 0.001) PBSCs (OR=1.8, *P* = 0.04) Post-engraftment: GVHD 2–4 (OR 4.8, *P* <0.0001), neutropenia (OR 3.4, *P* = 0.002), GCs (OR 3.2, *P* <0.0001), kidney failure (OR 3.8, *P* = 0.003), liver failure (OR 3.8, *P* <0.0001)	Pre-engraftment: Mort: 20.3% Att Mort: 14% OS in BSI versus no BSI (46.8% versus 64.1%, *P* <0.01) Post-engraftment: Mort: 17.2% Att Mort: 0 OS BSI versus no BSI (55% versus 63.3%, *P* = 0.25)
Avery *et al*. ^15^ * 2005 Cleveland Clinic	281 Allo-HSCT 1997–2003	No	No	-	-	4.3%		URD	Mort: 100% Att Mort: 10%
Matar *et al*. ^5^ 2006 MDACC	653 HSCT 2001		Stool culture weekly x3	4.7%	N/A	1.4%	28% (9/32)	-	Att Mort: 7%
Dubberke *et al*. ^23^ * 2006 BJH	968 auto- HSCT; 612 allo-HSCT 1996–2002	No *	Stool culture in patients tested for Cdiff	21%	-	3.9%	13% (42/334)	-	Mort: 53% Att Mort: 12.5%
Zirakzadeh *et al*. ^17^ 2008 Mayo Clinic	217 allo-HSCT 1998–2004	Yes	pre-HSCT swab or stool culture or PCR twice weekly and with diarrhea	10%	Cdiff, renal failure, AML, low platelet count, TBI, MAR	2.8%	27% (6/22)	Colo (27% versus 0%, *P*≤0.01)	Mort: 27.6% Mort (Colo): 45% Mort (non-Colo): 25% (HR 2.1, *P* = 0.028) Mort (Colo to BSI): 83%
Weinstock *et al*. ^12^ 2007 MSKCC	92 allo-HSCT 2004–2006	Yes *	Stool culture on admission for HSCT and also with diarrhea	40% Pre-HSCT n = 2, at HSCT n = 25, and post- HSCT n = 10	Acute leukemia, refractory anemia with excess blasts	15% (14/92)	4% (13/37) 27% (10/37) pre-HSCT	Colo (34% versus 1.8%, *P* <0.01)	Mort: 50% Att Mort: 14.3% Mort for VRE BSI versus other BSI (HR 5.1, *P* = 0.5) Mort (non-Colo) versus Mort (Colo non- progressors) (HR 0.8, *P* = 0.55)
Kamboj *et al*. ^7^ 2010 MSKCC	247 allo-HSCT 2008–2009	Yes *	Rectal swab culture weekly	27.5%		11% 13/23 (57%) were colonized	19% (13/68)	pre-HSCT Colo (OR=3.88, *P* = 0.005) T cell depletion (OR=10.89, *P* = 0.028)	Mort in VRE BSI versus non-VRE BSI versus no BSI 4.4% versus 15% versus 2% Att Mort: 9%
Vydra *et al*. ^18^ 2012 University of Minnesota	752 HSCT 491 adults 2004–2008	N/A	Perirectal swab cultures weekly	23% 6% (43/752) pre-HSCT	Leukemia MDS Age >60	8% **	14%	pre-HSCT Colo RR=3.3 ( *P* = 0.01) post-HSCT Colo RR=7.7 ( *P* <0.01) Engraftment delay acute GVHD 3–4	Mort **: 38% OS VRE BSI versus VSE BSI: 23% versus 48% ( *P* = 0.04)
Kang *et al*. ^10^ 2013 University of Chicago	152 HSCT 2008–2011	Yes	Rectal swab weekly cultures	100%			12.5%	vancomycin ( *P* = 0.017), prolonged neutropenia ( *P* = 0.001), IS ( *P* <0.001), VRE at week 1 ( *P* = 0.05) ***	Non-progressors versus progressors Mort: 4% versus 29% ( *P* = 0.001)
Tavadze *et al*. ^6^ 2014 Cleveland Clinic	800 Allo-HSCT 1997–2011	-	No	-	-	9.5% 17% (n = 13) previous VRE or Colo		Later year of HSCT (HR=1.06, *P* = 0.037) High HSCT-CS (HR=2.02, *P* = 0.022) ALL (HR=2.20, *P* = 0.003) URD (HR=2.75, *P* <0.001) UCB donor (HR=3.11, *P* = 0.003)	Mort: 96% Att Mort: 5% Worse OS: VRE BSI (HR=4.45, *p*<0.001) Year of transplant Male gender Older age Prior chemotherapies High HSCT-CS Lack of remission at HSCT URD CMV- positive donor
Satlin *et al*. ^13^ 2014 Weill Cornell University	238 allo-HSCT 287 auto-SCT 2007–2011	Yes	No	-	-	Allo-HSCT: 16.4% Auto-HSCT: 3.8%	-	Mismatched PBSCs (HR=3.76, *P* = 0.04) Time to engraftment (HR=1.06 per day, 95%, *P* = 0.005)	Mort: 18%
Ford *et al*. ^27^ 2015 Salt Lake City	300 auto-HSCT 2006–2013	Yes	Stool cultures on admission and weekly	36%	Lymphoma	3%	8.3% (9/108)	Colo	Mort: 0
Ford *et al*. ^8^ 2017 Salt Lake City	161 HSCT	Yes	Stool cultures on admission and weekly	pre-HSCT: 61% (66/109) day of HSCT: 43% (58/134)	Time from leukemia to HSCT Pre-HSCT Colo: RF of subsequent Colo (HR=3.8)	12% Pre- engraftment (10) Post- engraftment (9)	10% (at day 30) 12.5% (at day 90)	Pre-engraftment: Pre-HSCT Colo Colo at admission Post-engraftment: GVHD Pre-HSCT Colo	Pre-engraftment Mort: 20% Similar OS in VRE BSI versus other BSI Worse OS: All BSI (HR=3.6, *P* <0.006) Post-engraftment VRE BSI versus pre- engraftment VRE BSI (10% versus 80%, *P* = 0.0007) No influence of Colo on LOS or OS, trend to greater healthcare costs
Hefazi *et al*. ^9^ 2016 Mayo Clinic	203 AML Allo-HSCT 2004–2014	No	Perirectal or stool PCR on day 0 and twice weekly	Day 0: 36% Day 1–100: 10% >Day 100: 8%	HSCT-CS ≥3	Day 0–30: 5% 91% (10) were colonized Day 30–100: 0.4% (1) >Day 100: 4%	11% (10/88)	Age ≥60 ( *P* = 0.04) HSCT-CS ≥3 ( *P* = 0.03) Colo ( *P* = 0.003)	Mort: 55% (6/11), 9% within 100 days Att Mort: 0 Pre-HSCT VRE Colo no impact on outcomes/OS Colo after day 0 associated with worse survival (HR=2.2, *P* = 0.03)

*Studies in which all individuals developing febrile neutropenia were automatically started on empirical vancomycin **Specific data observed in adults ***Colonization, prior VRE, or delayed engraftment were not risk factors

VRE have often been considered an organism of limited virulence ^14^. However, data suggest that VREB may be associated with severe presentations in HSCT recipients, with, in at least some reported experiences, high rates of septic shock ^7, 13, 15^. Mortality estimates have been widely variable, ranging from 4–100% ^11– 13, 15– 18^. The most common manifestation of VRE infection in HSCT recipients is bacteremia—often catheter associated—usually occurring in the early post-transplant and peri-engraftment period, in the setting of severe mucositis and bacterial translocation ^8– 10, 12, 13, 15^. Other presentations include infections of the urinary tract, soft tissue, intra-abdominal space, and biliary tract as well as endocarditis and, rarely, infections of the central nervous system ^10, 19– 21^.

### Vancomycin-resistant enterococci colonization and vancomycin-resistant enterococci bacteremia and their impact on hematopoietic stem cell transplant outcomes

Patients with hematologic malignancies, especially those who undergo HSCT, are at a particularly high risk for VRE colonization and subsequent infection ^12, 17, 22^. Areas of controversy in which there are conflicting data are the association between pre-HSCT VRE colonization and the risk of VREB ^10, 12, 18^ as well as the effect VRE colonization and bacteremia have on HSCT-associated mortality ^6, 15, 17, 23^.

The frequency and impact of the progression from colonization to bacteremia is still not well understood. Studies report varying rates of such progression in the early post-HSCT period that range from 10–34% ^5, 7– 9, 12, 18^, together with mortality rates that range from 40–100% ^12, 17^. This variability may result from differing severity of underlying illness in largely heterogeneous transplant populations as well as different screening and treatment strategies, changing epidemiology of VRE colonization across transplant centers, and evolving transplant practice and supportive care measures over the last two decades. In addition to these varied reported experiences, while some authors have reported that VRE colonization and/or BSI are independent risk factors for mortality ^12, 18, 24^, others have argued against causality and conclude that these are simply markers of a complicated post-transplant course ^6, 23, 25, 26^.

Clarification of the relationship between VRE colonization and the risk of subsequent VREB in these patients is necessary to accurately inform decisions related to the use of empirical or pre-emptive VRE-active therapy in HSCT recipients.

### Vancomycin-resistant enterococci colonization: prevalence and risk factors

The prevalence of VRE colonization in HSCT recipients has increased over time. In 2001, 4.7% of HSCT recipients at the M.D. Anderson Cancer Center were colonized ^5^. During the period of 1998–2004, VRE was present in 10% of individuals admitted for HSCT at the Mayo Clinic, Rochester ^17^. In the same center, the prevalence nearly quadrupled in the following decade ^9^. During the 10-year study period, all 203 allogeneic HSCT recipients were screened for fecal, perianal, or perirectal VRE colonization by PCR testing of colonial growth on blood agar plates at the time of admission for HSCT and with subsequent twice-weekly surveillance. VRE was detected prior to transplantation in 73 (36%) patients, while 21 (10%) were newly colonized in the first 100 days post-HSCT and 107 (53%) remained uncolonized. Those colonized at the time of admission for HSCT had a higher comorbidity index compared to non-colonized ( *P* = 0.02) individuals. Comparison of the periods before and after the introduction of PCR screening in 2009 revealed no differences in rates of colonization and were in overall agreement with other contemporary studies that found a prevalence of 23–40% ^7, 9, 12, 18^.

To analyze the relationship among VRE colonization, bacteremia, and post-transplant mortality, Ford and colleagues studied 161 patients with acute myeloid, leukemic, or biphenotypic leukemia who underwent HSCT between 2006 and 2014, making a distinction between colonization during the preparative period before HSCT and colonization detected immediately before HSCT ^8^. In their cohort, 109 patients had weekly surveillance stool cultures before admission for HSCT. A total of 66 (61%) were VRE positive in the pre-HSCT period, with the greatest risk factor being the number of inpatient days in the interval between the initial admission for leukemia and the HSCT. One-third of these patients were no longer colonized at the time of admission for HSCT (58 of 134, 43%) but were at increased risk for subsequent colonization. Among those apparently re-colonized, the newly acquired strain differed from the original one more than half the time.

Fewer studies have examined VRE colonization and bacteremia in autologous HSCT recipients ^16, 27^. Ford and colleagues described their experience at the Intermountain Blood and Transplant Program, where 36% of 300 autologous HSCT recipients were colonized ^27^. Of these, 8.3% developed VREB, and all nine bacteremic patients were previously colonized. Neither VRE colonization nor bacteremia was associated with reduced overall survival (OS). A separate study conducted at Cornell ^28^ of 326 HSCT recipients (197 allogeneic, 129 autologous) determined that, compared to autologous HSCT recipients, patients with allogeneic HSCT were more likely to be colonized on admission for HSCT (28% versus 12%, *P* <0.001) and to become colonized during their transplant hospitalization (52% versus 20%, *P* <0.001).

The risk factors for VRE carriage in HSCT recipients are similar to those identified in cancer patients. Heavy antimicrobial exposure, severe underlying disease, and frequent and prolonged contact with the healthcare system are among the risks most commonly described in the literature ^8, 9, 12, 17, 18, 29– 31^. More than their simple presence, the density of *enterococci* in the GI tract plays a key role in VRE colonization and the susceptibility to VREB ^14^. *Enterococci* constitute a small proportion of the gut microbiota ^32^. Under exposure to antibiotics with broad-spectrum Gram-negative (GN) and GP bacteria coverage, shifts in the intestinal flora facilitate enterococcal dominance. When stimulated via Toll-like receptors by flagella and lipopolysaccharide of GN rods and anaerobes, Paneth cells in the GI tract produce REGIIIγ, a C-type lectin with antimicrobial activity against GP bacteria ^33, 34^. Depletion of GN rods under antibiotic pressure results in enterococcal overgrowth. Researchers at the MSKCC recently showed that the administration of metronidazole, neomycin, and vancomycin allowed VRE to become the predominant intestinal species in mice, remaining for up to two months after antibiotic discontinuation. In humans, VRE invasion of the bloodstream was preceded by its predominance in the GI tract ^35^. In a later study, enterococcal domination increased the risk of VRE BSI by ninefold ^36^. *Clostridium difficile* infection and its treatment with oral metronidazole or oral vancomycin ^37^ have also been linked to the development of VRE colonization and infection. These two infections share epidemiological features and, mechanistically, may occur under antibiotic pressure, possibly through the inhibition of Paneth cell secretion of alpha-defensins, resulting in their co-occurrence as a frequent phenomenon ^17, 38, 39^.

### Impact of hematopoietic stem cell transplant colonization on vancomycin-resistant enterococci bacteremia

VRE colonization before and after HSCT is associated with an increased risk of VRE infection ^12, 17^ and has been found to be an independent risk factor for VREB in several studies ^7, 9, 18^ ( Table 1).

Ford and colleagues analyzed the impact of colonization detected at different time points pre-transplantation—during the period between leukemia diagnosis and HSCT and at the time of admission for HSCT—and post-HSCT on the incidence of pre- and post-engraftment bacteremia. Patients colonized before HSCT had an increased risk of HSCT-associated VRE infections (32% versus 7%, *P* = 0.001), including bacteremia during both pre-engraftment (9%) and post-engraftment (9%) periods, while none were observed in patients without prior colonization. Pre-engraftment bacteremia was also more common in the cohort of patients colonized at the time of admission ( *P* = 0.03). In contrast, new onset of colonization at a later time was not a predictor of bacteremia.

While the prevalence of colonization has increased in recent years, the frequency of progression to VREB in colonized patients appears to have decreased from 27–34% in early studies ^8, 9, 10, 18^ to 10–15% according to more recent data ^12, 17, 40^.

Identifying the risk factors that contribute to the development of subsequent VREB in colonized HSCT recipients might enable the prediction of patients who could benefit from early empirical anti-VRE antibiotic therapy in the case of suspected GP bacteremia.

This specific question was addressed in a retrospective chart review of patients who received allogeneic HSCT from 2008 to 2011 ^10^. VRE colonization was tested with weekly rectal swab cultures until confirmed to be positive. Neutropenic patients received antibacterial prophylaxis with moxifloxacin and, when febrile, received vancomycin intravenously according to guideline recommendations, although adherence was less than complete. Of 152 colonized patients, 19 (13%) patients subsequently developed VREB. Risk factors for progression to bacteremia included the use of vancomycin after VRE detection ( *P* = 0.017), prolonged duration of neutropenia (>30 days) ( *P* = 0.001), immunosuppression ( *P* <0.001), and timing of first VRE surveillance screen positivity at week one from HSCT ( *P* = 0.005). Interestingly, VRE colonization during a previous admission was not an independent risk factor for bacteremia ( *P* = 1.0).

A study conducted at the Mayo Clinic revealed that 10 (91%) of the 11 patients who developed VREB in the 30 days following HSCT were previously colonized as determined by PCR in stool or perirectal swab. Older age (>60 years) (hazard ratio [HR] 5.1 [1.0–34], *P* = 0.04), high HSCT-associated comorbidity index (HR 4.6 [1.1–24], *P* = 0.03), and VRE colonization (HR 15 [2.7–299], *P* = 0.003) were independent risk factors for the development of VRE BSI ^9^. The overall rate of progression from colonization to bacteremia was 11%. In colonized patients with febrile neutropenia (FN), it was 21%. Moreover, in FN patients with GP bacteremia, VRE was eventually identified in 67% of cases in the VRE-colonized but in only 25% of the non-colonized patients. Thus, rapid identification of colonized patients with the highly sensitive PCR testing ^41^ may prove useful in dictating appropriate initial antibiotic therapy in the febrile neutropenic HSCT recipient. Nevertheless, the use of PCR in the detection of VRE has diagnostic limitations ^42– 44^. The detection of *vanB* sequences carries a high false-positivity rate, owing to the presence of *vanB* genetic elements in intestinal non-enterococcal bacteria, notably in anaerobes such as *Clostridium* spp. and *Streptococcus* spp. ^45^. In a recent analysis of the performance of the GeneXpert® *vanA/vanB* assay (Cepheid AB, Solna, Sweden), PCR had a sensitivity of 87.1%, a specificity of 99.7%, and positive and negative predictive values of 98.0% and 97.7%, respectively. However, the *vanB* PCR had a considerably lower specificity of 77.6% and a PPV of 0.4 ^46^. Therefore, in institutions in which *vanB* VRE is prevalent, the detection of VRE by PCR may require confirmatory testing by culture.

### Other risk factors for vancomycin-resistant enterococci bacteremia

Individuals with hematological malignancies are frequently hospitalized, often for long periods of time, needing central venous access and often requiring ICU level of care, all factors that, in addition to immunosuppression and neutropenia, further increase their risk of VREB. Prolonged and repeated antibiotic exposures, in particular to ceftriaxone and metronidazole, have been associated with VRE colonization and infection in multiple patient populations, including HSCT recipients. Previous studies showed that vancomycin exposure increased the risk of developing VRE infection ^47– 51^. Taur and colleagues reported that shifts in the intestinal microbiota towards VRE domination and bacteremia were more likely to occur with the use of metronidazole compared with β-lactams or vancomycin ^36, 52^. In a study by Satlin *et al*., VRE did not cause any of 101 BSIs in neutropenic patients not receiving antibacterials but caused 32 (55%) of 58 BSIs in neutropenic patients receiving a broad-spectrum β-lactam agent, especially meropenem ( *P* <0.001) ^13^. The investigators concluded that the development of GP bacteremia in the setting of broad-spectrum antibiotic use warrants the addition of antibiotics active against VRE, whereas a first episode of FN with bacteremia in a patient not receiving antibiotics should not prompt empiric VRE coverage. In a later study, they suggested that the latter group could benefit from empirical VRE coverage if prior colonization is documented ^28^.

Immunosuppression and disruption of the intestinal mucosa facilitate bacterial translocation in HSCT recipients ^53^. Prolonged neutropenia and mucositis in the peri-engraftment period and, occurring later, graft-versus-host disease (GVHD) predispose these patients to the development of VREB ^8, 13, 18^. Paneth cells have a key role controlling the inflammatory response to pathogens in the small bowel and maintaining its commensal flora. In GVHD, the loss of Paneth cells facilitates bacterial translocation ^54– 56^.

In a 2012 study, the risk of developing VREB increased with each week’s delay in engraftment, rising from 4.5% before day 21 to 15% between day 36 and 42, and was highest for those not engrafted by day 42 ^18^. Umbilical cord blood transplant recipients in whom engraftment is particularly delayed appear to be at higher risk ^6^ of VREB.

In a recent publication, Webb *et al*. proposed an integrative scoring system for the prediction of VREB in HSCT recipients ^57^. The risk factors in the model included VRE colonization with highest score weight, severe neutropenia, GI disruption, renal insufficiency, and the use of antibiotics (anti-anaerobic, carbapenem, aminoglycoside, and cephalosporin). A score greater or equal to five points would identify patients at high risk of VREB with 77% sensitivity and 79% specificity. More importantly, compared to using colonization status alone, the use of the scoring system would have resulted in a 43.2% reduction of anti-VRE antibiotic use.

### Impact of vancomycin-resistant enterococci bacteremia on hematopoietic stem cell transplant outcomes

Early studies reported 100-day mortality rates as high as 83–100% ^6, 12, 15, 17^ and attributable mortality rates of 8–14% ^7, 11, 12, 15^ in HSCT recipients with VRE BSI ( Table 1).

VREB occurring early post-HSCT, especially in the pre-engraftment period, has been associated with poor outcomes and, in some studies, is a risk factor for worsened survival ^6, 11, 18^. In their report of cases of early VREB, all occurring within 21 days of HSCT, Avery *et al*. observed that all 10 patients died within 73 days, despite appropriate therapy and likely source control (indwelling catheter). Only one death, however, was attributed to VRE infection in this cohort of patients with multiple comorbidities and coinfections ^15^.

In contrast, Ford *et al*. observed that patients with pre-engraftment VREB had a relatively good prognosis, with one-year survival of 80%. Although pre-engraftment bacteremia with any organism was associated with worse OS, risk factors and survival outcomes in patients with pre-engraftment VREB did not significantly differ when compared to those in patients with pre-engraftment bacteremia due to other organisms, including vancomycin-susceptible *enterococcus* (VSE) ^8^. The group at the Mayo Clinic ^9^ observed that only one of the six patients with VREB and fatal outcome died within the first 100 days post-HSCT and no death was directly related to VRE infection. This lower mortality occurred despite delayed use of anti-VRE antibiotics (median two days after the onset of bacteremia) in 7(63%) of the 11 patients with VRE BSI. This observation is in line with the findings of Ford and colleagues and others ^8, 23, 25^ and suggests that the effects of VRE may not be related to its virulence but instead that VREB may be a surrogate marker of comorbidity burden and poor overall status. Mortality is likely associated with the severity and duration of immunosuppression and other comorbidities.

In contrast to pre-engraftment VREB, post-engraftment VREB carried higher morbidity and mortality in the study by Ford *et al*. Compared to pre-engraftment VREB and bacteremia with other organisms, post-engraftment VRE BSI carried the highest mortality and healthcare costs. However, patients with post-engraftment bacteremia had significant life-threatening comorbidities (severe GVHD, leukemic recurrence, and multi-organ failure).

### Impact of vancomycin-resistant enterococci colonization on hematopoietic stem cell transplant outcomes

The impact of VRE colonization on VRE BSI and HSCT outcomes has been assessed with some relative degree of consensus in recently conducted studies. The group in Utah (Ford *et al*.) observed that pre-HSCT colonization was associated with an increased incidence of subsequent colonization and of HSCT-associated VREB but not with worse survival or increase in length of stay (LOS) ^8^ ( Table 1).

In line with these findings, Hefazi *et al*. ^9^ noted that pre-transplant VRE colonization was associated with increased rates of VRE BSI (11% versus 3%, *P* = 0.03) and hospitalization (85% versus 71%, *P* = 0.02) within 100 days of HSCT and was an independent risk factor for these outcomes (HR 3.9 [1.0–17], *P* = 0.04 and HR 2.3 [1.1–5.4], *P* = 0.02, respectively). However, it had no significant impact on the incidence of FN, BSI with other bacteria, ICU admission, mortality (10% versus 7%), or OS, suggesting VRE colonization may only be a surrogate marker for poor outcomes. In contrast, VRE colonization occurring within the first 100 days after HSCT was an independent risk factor for worse OS when compared to those who remained free of colonization. This group also had higher rates of death due to relapse (29% versus 9%, *P* = 0.03) and chronic GVHD (19% versus 6%, *P* = 0.07). In patients with FN, however, OS was not affected by the presence of VRE colonization. These two studies are in agreement with others ^10, 12^ that found that pre-transplant VRE colonization had no impact on post-HSCT survival in the absence of occurrence of VRE BSI, but they contrast to the report of Zirakzadeh *et al*. ^17^, who reported that colonization was an independent risk factor for 100-day post-HSCT mortality.

In their examination of the gut microbiota in HSCT patients, Taur and colleagues at the MSKCC demonstrated that microbial diversity at the time of stem cell engraftment predicts HSCT survival ^58^. Decreased diversity and intestinal domination by *enterococc*i not only correlates with the spectrum of antibiotics administered to patients but also predicts reduced survival. Ongoing studies are investigating whether reconstitution of intestinal microbiota using fecal microbial transplantation following allogeneic HSCT may provide an approach to optimize clinical outcomes and survival (clinicaltrials.gov identifier: NCT02269150).

Thus, among allogeneic HSCT patients, VRE colonization is a risk factor and a precondition for VREB, especially when present in the GI tract as a dominant organism. VREB is associated with decreased survival (especially post-engraftment) but not with attributable mortality, with death largely related to comorbidities. Autologous HSCT patients may have a prevalence of VRE colonization similar to that seen in allogeneic HSCT patients, but they appear to have a lower risk of bacteremia without associated increased risk of mortality.

## Prevention of vancomycin-resistant enterococci acquisition and infection

With the emergence of VRE as a leading cause of BSI in HSCT recipients, as well as healthcare-associated infections in general, preventive strategies emerge as the key to controlling the spread of VRE infection in HSCT units. Prevention activities usually combine hand hygiene, environmental cleaning/disinfection, contact isolation, and surveillance ^59^. There is, however, variation across centers in the application of infection control measures for HSCT recipients ^60^.

### Cleaning and decontamination

Hand hygiene programs paired with feedback systems including electronic surveillance have been demonstrated in hematology units to increase compliance and to significantly reduce the nosocomial transmission of VRE ^61, 62^.

In an Australian study conducted in high VRE risk wards, the incorporation of bleach-based disinfection markedly reduced VRE environmental contamination by 66.4% ( *P* = 0.012) and also resulted in lower rates of newly acquired VRE as well as VREB by 25% ( *P* = 0.016) and 83% ( *P* <0.001), respectively ^63^. Daily bathing with 2% chlorhexidine (CHG) has been proposed to prevent bacterial colonization in critically ill patients, and its impact has been studied particularly in ICU patients, in whom CHG has been shown to reduce VRE burden ^64– 66^.

Limited and controversial data are available in HSCT patients. Although one study failed to show any benefit of use of CHG wipes in hematology-oncology patients ^67^, a contemporaneous multicenter randomized trial including HSCT patients demonstrated a 25% ( *P* = 0.05) reduction in acquisition of VRE colonization but no significant reduction in VREB ^68^. In a quasi-experimental study, Mendes and colleagues evaluated the impact of prolonged exposure to CHG (using shower bath and liquid soap formula) on the incidence of VRE colonization and of infection in a BMT unit and found a significant ( *P* = 0.001) reduction in the rate of both ^69^. This occurred despite the absence of a substantial effect on overall hospital rates of VRE or on the incidence of infection and colonization with multidrug-resistant (MDR) GN bacteria ^70^.

Novel methods for environmental decontamination have emerged with promising results, including the use of hydrogen peroxide vapor ^71^, copper alloy surfaces ^72^, or pulsed-xenon ultraviolet room disinfection ^73^. These techniques are currently being evaluated in an ongoing trial with HSCT recipients (clinicaltrials.gov identifier: NCT02463214).

### Surveillance strategies

The benefit and cost effectiveness of routine VRE surveillance, as well as the optimal testing frequency, are controversial and it has not been universally adopted in HSCT units. The Society for Healthcare Epidemiology of America (SHEA) as well as the American Society of Bone Marrow Transplantation (ASBMT) infection prevention guidelines from 2009 limit their relevant recommendation to the following: “VRE rectal or stool active surveillance cultures to identify colonized patients can be considered if there is evidence for ongoing transmission of VRE on a HCT unit” ^59, 74^.

Nonetheless, pre-HSCT screening can identify a high proportion of patients at risk of VREB. The variability in sample collection technique and poor sensitivity of culture-based methods for the detection of VRE is well recognized ^75^, but few studies have evaluated PCR techniques in HSCT recipients. In centers using PCR, up to 90–100% of patients developing bacteremia had previously been colonized ^7, 9, 17^. Hefazi *et al*. also showed that the likelihood of VRE being the etiologic organism of GP bacteremia in FN patients was 2.5 times higher in patients previously colonized, arguing for surveillance to guide empirical therapy in FN patients ^9^.

### Barrier precautions and isolation

Contact precautions (CPs) against VRE include the use of single-patient rooms and of gowns and gloves during patient contact ^59^. Barrier precautions and contact isolation have, however, not been clearly shown to be effective at preventing VRE colonization ^29, 76^. Nevertheless, these are widely used in HSCT recipients, based on extrapolation from the standard practice for containment of other resistant bacteria rather than direct evidence. Literature has provided evidence supporting the use of CPs for the prevention of resistant bacteria including VRE in patients with hematologic malignancies ^77, 78^, albeit there are concerns for biased reporting of positive results and studies that often evaluate multiple interventions ^8^. The systematic implementation of CPs has also been questioned for lack of proven efficacy and collateral adverse effects including associated stigma leading to poorer patient care ^79– 82^. Two cluster-randomized trials evaluated the efficacy of active surveillance for MRSA and VRE colonization and the universal use of gowns and gloves (compared to targeted use in colonized patients): neither intervention resulted in a difference in MRSA and VRE acquisition ^76, 83^. More than 40 hospitals in the United States have abandoned the implementation of universal CPs. Elimination of this measure has resulted in no change in rates of VRE infection in several recent studies and could also result in significant cost saving and decreased healthcare worker time ^84– 86^. Selective use of CPs for patients at high risk of transmission—for instance, those with draining wounds or diarrhea—or for healthcare professionals performing high-risk patient care activities may be more efficient ^87^.

VRE-colonized patients usually stay colonized for an extended period of time once initially detected. HSCT recipients with previous VRE colonization or infection should continue to receive CPs during hospital readmissions. Compliance can be facilitated by the use of electronic health records programmed to provide alerts ^59^. However, data providing guidance regarding the optimal duration of precautions for HSCT recipients with a history of VRE are lacking. Criteria for discontinuation of CPs is determined according to individual institutional protocols (e.g. three consecutive sets of screening cultures negative for VRE obtained on separate days for a patient not on effective anti-VRE agents) ^74^.

Almyroudis and colleagues recently evaluated the impact of discontinuing systemic surveillance and CPs on the incidence of vancomycin-resistant *Enterococcus faecium* bacteremia in patients with hematologic malignancies and HSCT recipients ^88^. During the first period of the study (2008–2011), the local VRE prevention protocol included weekly fecal surveillance of all patients admitted to the hospital and lifelong contact isolation of colonized patients. However, the authors found that neither colonization nor bacteremia incidence was lowered by strict implementation of these measures ^29^. Moreover, they did molecular analysis of fecal and blood isolates for genetic similarity to define clonality and to identify common sources of infection or modes of transmission to find a primarily sporadic pattern of VRE acquisition in which the majority of the patients harbored unique VRE strains. Discontinuation of strict precautions and surveillance did not affect the incidence of VREB over the following three-year period. They also observed that the use of levofloxacin prophylaxis during neutropenia and daily CHG bathing had no effect on either ( *P* >0.05). The use of antibiotics, incidence of MRSA bacteremia, and *C. difficile* infection remained stable during the two time-periods ( *P* >0.05).

### Modification of microbiota

In the past, selective digestive tract decontamination has been used in hematology and ICU units to prevent GN bacteremia ^89^. An opposite strategy aiming to restore microbiota diversity using autologous fecal microbiota transplantation is being explored in clinical trials to prevent breakthrough infections with dominant MDR organisms like VRE (clinicaltrials.gov identifier: NCT03061097). The anecdotal use of short courses of linezolid or daptomycin paired with other decolonization methods—including the use of polyethylene glycol for bowel preparation to wash-out the fecal bacterial population prior to the administration of antibacterials—has not been tested in large cohorts of HSCT recipients ^90^.

### Prophylactic antibiotics

The rate of progression from VRE colonization to bacteremia in the early post-transplant period and the high associated mortality in earlier studies have led to considerations of administration of prophylactic antibiotics at the time of transplantation. Wong *et al*. demonstrated only temporary (for up to two weeks) suppression of GI VRE colonization with the investigational non-absorbable glycolipodepsipeptide ramoplanin ^91, 92^. The addition of systemic anti-VRE agents such as linezolid or daptomycin to the peri-transplant prophylactic regimen is an alternative intervention but with non-negligible associated risks, including emergence of resistances ^93, 94^, and associated toxicities, notably linezolid-induced cytopenias. The 2009 ASBMT guidelines for the prevention of infections in HSCT recipients recommends against the use of anti-GP agents for prophylaxis ^74^.

### Antibiotic stewardship

Antibiotic exposure is a key risk factor for VRE colonization and infection in hematology patients ^29, 35, 95^. Antimicrobial stewardship programs (ASPs) are therefore crucial as a complement to infection control strategies ^96, 97^.

The precise association between vancomycin use—both intravenous and oral—and VRE colonization and infection remains unclear owing to conflicting data from animal models and clinical studies ^35, 36, 50, 52, 98, 99^. Al Nassir and colleagues observed that VRE-colonized patients treated for diarrhea due to *C. difficile* with oral vancomycin (and/or metronidazole) had persistence of VRE intestinal overgrowth ( *P* <0.049) ^37^. In a meta-analysis by Carmeli *et al*. of 15 studies with optimal control groups, vancomycin exposure conferred a 2.7-fold increased risk of VRE acquisition ^100^. However, a subsequent individual case-control study by the same group failed to show such an association ^49^. Therefore, the effect of restricting vancomycin on acquiring clinically significant VRE at the population level is still unclear. A large ecologic study found that vancomycin was the most significant 'modifiable' risk factor resulting in VRE colonization ^50^, yet a systematic review was not able to conclude that restriction of vancomycin prescribing had any effect on the prevalence and incidence of VRE colonization and infection in US hospitals ^101^. Nevertheless, as demonstrated by Taur *et al*. ^36^, intestinal domination of VRE driven by antimicrobial pressure precedes bacteremia. To prevent this, the ASBMT guidelines of infection prevention recommend minimization of the use and duration of vancomycin, agents with anti-anaerobic coverage (e.g. metronidazole), and third-generation cephalosporins ^74^. Restricted use of broad-spectrum antibiotics and early implementation of targeted therapies ^96, 97, 102^ can effectively reduce VRE overgrowth, colonization, and subsequent infection.

## Treatment

### Daptomycin

The current treatment of invasive VRE infections primarily revolves around the use of either daptomycin or linezolid. Daptomycin has the potential advantages of having bactericidal activity against many strains of VRE as well as a relative lack of toxicity and of drug–drug pharmacokinetic (PK) interactions. Its disadvantages include ready selection of non-susceptible strains and high acquisition cost.

Reduced susceptibility of VRE to daptomycin is an increasingly encountered phenomenon and may occur subsequent, or during exposure, to the antibiotic in the clinical setting ^103^. Thus, DiPippo and colleagues found that exposure to daptomycin within the prior 90 days was associated with a significant risk that subsequent *E. faecium* bacteremia will be due to a daptomycin non-susceptible (DNS) strain ^104^. Furthermore, *in vitro* studies found that, with commonly used doses of <10 mg/kg/day, free drug Cmax values usually fall within the mutant selection window ^105^. In addition, many isolates with MICs of 3-4 mcg/mL (isolates with MIC >4 mcg/mL are non-susceptible) carry mutations, especially in *liaFSr*, that are associated with loss of daptomycin bactericidal activity ^106^, and exposure of these strains to daptomycin concentrations consistent with administration of ≤10 mg/kg/day allowed regrowth ^107^.

In a prospective cohort study of a general patient population, high-dose (≥9 mg/kg/day) daptomycin administration was associated with greater survival than was lower-dose (6–9 mg/kg/day) administration ^108^. Similarly, a national retrospective cohort study in the Veterans Administration system found that doses of ≥10 mg/kg/day were associated with improved survival relative to doses of 8 and 6 mg/kg/day ^109^. In contrast, Shukla and colleagues found, in a multicenter retrospective cohort study, that, while an MIC of 3–4 mcg/mL (by ETest, but not by standard broth microdilution) was an independent predictor of microbiologic failure, initial dosing of daptomycin of ≥8 mcg/mL did not improve outcomes ^110^. Of note is that the only independent risk factor for failure other than MIC of 3–4 mcg/mL was immunosuppression, which was present in 48 of the 62 patients with vancomycin-resistant *E. faecium* bacteremia; 26 of the 48 were neutropenic and 17 were organ transplant recipients.

In a smaller retrospective single-center study limited to HSCT patients and/or patients with hematologic malignancies with VRE (all *E. faecium*) bacteremia, daptomycin ETest MIC of 3–4 mcg/mL, when compared to lower MICs, with regard to 30-day all-cause mortality inexplicably had an adjusted HR of 0.27 (95% confidence interval [CI] 0.07–1.06, *P* = 0.06) ^111^. Treatment with daptomycin doses >6 mg/kg/day (median dose of 8.1 mg/kg/day) was not significantly associated with less microbiological failure, but the number of patients for whom dosing data were available was limited.

These studies, taken as a whole, suggest, but do not clearly demonstrate, that daptomycin doses of 6 mg/kg/day may be therapeutically inadequate and may increase the likelihood of selection of resistant mutants and that higher doses (e.g. 10 mg/kg/day) may be preferable.

### Linezolid

Linezolid has excellent oral bioavailability but is bacteriostatic, has potential toxicity (including hematologic), and also has a number of important interactions with other drugs.

However, the distinction between bacteriostatic and bactericidal activity of an antibiotic is, to some extent, arbitrary ^112^. Furthermore, while many clinicians believe that bactericidal activity is preferred in bacteremic patients with neutropenia, evidence to support this appears to be lacking ^112^, with the exception of endocarditis.

Although prolonged linezolid therapy in excess of 14 days carries a risk of development of cytopenias, particularly thrombocytopenia, in all patient groups, its effects in patients recovering from chemotherapy-induced cytopenias raise particular concern. A series of studies have examined the hematologic safety of linezolid in chemotherapy-induced neutropenia including in the pre-engraftment period of HSCT. A retrospective analysis of 43 HSCT and hematologic malignancy patients with VREB (42 due to *E. faecium*) treated with either linezolid (n = 29) for a median of 11.5 days or daptomycin (n = 43) for a median of 13.0 days found no significant difference in outcomes or in duration of thrombocytopenia or neutropenia ^113^. In a retrospective analysis of patients receiving induction chemotherapy for newly diagnosed acute myelogenous leukemia, the median times to neutrophil recovery in those who received ≥14 days of linezolid or vancomycin were 29 days and 26 days ( *P* = 0.487), respectively ^114^. This was true despite the fact that linezolid was administered for a duration of 27 days compared to 16 days ( *P* <0.001) of vancomycin administration. In a randomized trial, patients with FN received either linezolid or vancomycin for GP coverage; these were administered for means of 11.4 days and 11.5 days, respectively ^115^. While there was no difference in time to platelet recovery, neutrophil recovery was modestly delayed. Finally, no significant difference was found in time to engraftment in allogeneic HSCT recipients who received either linezolid for a median of 14 days (range: 7–34 days) or vancomycin for a median of 16 days (range: 8–33 days) ^116^. In a study of 100 HSCT and hematologic malignancy patients with fever and neutropenia, 35 of whom received linezolid beginning after persistence of FN for 48 hours while the remainder continued to receive a glycopeptide, severe neutropenia occurred significantly less frequently in the linezolid recipients ^117^.

Resistance to linezolid may occur, especially after prolonged exposure, but remains quite infrequent. Thus, surveillance programs found that only 0.78% of *Enterococci* isolates in the US and 0.22% worldwide were linezolid resistant ^118^.

### Linezolid versus daptomycin: targeted therapy

Recent reports provide conflicting results regarding the relative efficacy of linezolid and daptomycin in the treatment of VREB in general hospital populations.

A retrospective analysis of patients with VREB treated at Veterans Administration Medical Centers (VAMC) across the country from 2004–2013 examined outcomes in 319 patients treated with linezolid and 325 given daptomycin ^119^. There was a statistically significant relationship between the use of linezolid and treatment failure (adjusted relative risk [RR] 1.15, 95% CI 1.02–1.30, *P* = 0.026), as well as 30-day mortality and microbiologic failure.

In a retrospective national VAMC study of 2,630 evaluable patients with VREB, one-fifth of whom were included in the study described above ^119^, linezolid therapy (n = 1,348) was associated, after matching by propensity score, with increased mortality relative to daptomycin (n = 1,055) treatment (RR 1.13, 95% CI 1.02–1.26, *P* = 0.015) ^120^. In addition, the 227 patients initially given linezolid but whose therapy was changed to daptomycin also had lower mortality than those treated with only linezolid (RR 1.29, 95% CI 1.03–1.63, *P* = 0.021). Daptomycin treatment was associated with significantly lower mortality only in patients with endocarditis (6.6% of the total cohort). Only a minority of patients were significantly immunocompromised, including those who had received solid-organ transplant (2.7%), those with hematologic malignancy (15.9%), and those with neutropenia (7.9%). The median durations of bacteremia in daptomycin and linezolid recipients were three days and two days ( *P* <0.001), respectively. An important potential confounder in this analysis was the potential role of daptomycin synergy with β-lactam antibiotics, which were received by approximately four-fifths of patients.

In contrast to the VAMC studies, two recent meta-analyses reached very different conclusions. A meta-analysis that included 11 retrospective cohort studies available by November 2015 with a total of 1,339 patients with all daptomycin recipients receiving ≥6 mg/kg/day found no significant differences in overall crude mortality, clinical cure, microbiological cure, or incidence of relapse when compared to linezolid in the treatment of VREB ^121^. The authors pointed out that the individual studies were heterogeneous and had relatively small sample sizes.

Another meta-analysis of 13 studies published before 1 January 2014 that included 532 daptomycin recipients and 656 given linezolid found that daptomycin therapy was associated with greater mortality (odds ratio [OR] 1.43, 95% CI 1.09–1.86, *P* = 0.009) ^122^. There was, however, no significant difference in microbiological cure. Heterogeneity of included studies was detected and all studies were retrospective with relatively small sample sizes.

Comparative data related to neutropenic and/or HSCT patients is extremely limited. In a single center retrospective analysis of 72 HSCT and hematologic malignancy patients with VREB, 42 of which were due to *E. faecium*, 43 received daptomycin (median dose: 4.5 mg/kg every 24–48 hours) and 29 received linezolid ^113^. There was no significant difference in success rates. Similarly, Patel and colleagues reported their single-center retrospective analysis of adult oncology patients with VREB, with 32 and 33 having received daptomycin and linezolid, respectively ^123^. Of the total of 65 patients, 36 (55.4%) had acute leukemia, 11 (6.9%) were HSCT recipients, and 42 (64.6%) were neutropenic at the start of antibiotic therapy. Microbiological cure was achieved in 22 (71%) daptomycin recipients and 26 (75.6%) of those who received linezolid, while 8 of the 33 (25.8%) of the former and 7 of the 32 (20.6%) of the latter died.

### Empiric therapy

The 2010 IDSA guidelines recommend that consideration be given to modification of the initial empiric therapy for patients who are at risk of VRE infection with risk factors including colonization or prior infection with the organism as well as the presence of ‘high endemicity’ of VRE within the treating institution ^102^. In such circumstances, they recommend the early administration of either linezolid or daptomycin. The 2016 ESMO guidelines do not address the issue ^124^.

Lisboa and colleagues retrospectively examined outcomes in 100 VRE-colonized HSCT and hematologic malignancy patients with fever and neutropenia to determine the effect of empiric linezolid administration on outcome ^117^. The policy at their institution was to consider the use of linezolid in colonized patients with persisting fever after 48 hours of administration of an antibiotic with broad-spectrum GN coverage plus a glycopeptide. The latter was discontinued when linezolid was prescribed. A total of 14 episodes of VREB subsequently occurred in the 65 patients who continued to receive a glycopeptide, while none occurred in the 35 patients switched to linezolid. The pre-emptive administration of linezolid, however, had no significant effect on overall mortality.

### Other antibiotics

***Quinupristin/dalfopristin*.** In 2010, FDA approval of quinupristin/dalfopristin for the treatment of bacteremia due to *E. faecium* was withdrawn because of lack of evidence of efficacy.

***Tedizolid*.** Tedizolid shares its mechanism of action with linezolid but may be more potent ^125– 127^. Its capability of interacting with the 23S ribosomal subunit with higher affinity allows it to maintain activity even in the presence of target site modifications conferring linezolid resistance. Isolates with the chloramphenicol-florfenicol resistance ( *cfr*) gene retain tedizolid MICs <4 mg/L ^128^. MICs for tedizolid are frequently fourfold to eightfold lower than those of linezolid, and, although not routinely reported, can be inferred from linezolid MIC values ^129^. From the PK standpoint, tedizolid has a more favorable profile. With a longer half-life and oral bioavailability above 90%, it can be given once daily ^126^, orally or intravenously, and does not require dose adjustments with renal or hepatic impairment ^130^.

In two phase III clinical trials, ESTABLISH-1 and ESTABLISH-2, tedizolid was non-inferior to linezolid for the treatment of acute bacterial skin and skin structure infections (ABSSSIs) ^131, 132^. Patients receiving “long-term systemic immunosuppressive therapy” were excluded. These studies led to the drug’s FDA approval for this indication in 2014. The use of tedizolid in systemic VRE infections has not, however, been evaluated. Tedizolid may offer a better side effect profile than does linezolid. Prolonged oxazolidinone therapy is associated with toxicities, some of which derive from impairment of protein synthesis at the mitochondrial level, including lactic acidosis and both peripheral and optic neuropathy, but most commonly myelosuppression ^133^. Some data suggest a lack of association of tedizolid with impaired eukaryotic mitochondrial function ^134^. With the potential for lesser myeloid toxicity, tedizolid may be beneficial for long-term therapy, especially in patients with hematological malignancy. A recent study examined the hematological effects of 21 days of treatment with different doses of tedizolid (200 mg, 300 mg, and 400 mg daily) compared to standard linezolid doses (600 mg twice daily) and placebo in groups of eight patients. Progressive tedizolid-induced thrombocytopenia occurred in a dose-dependent manner, and the effects of tedizolid at 400 mg daily were similar to those of linezolid. No adverse platelet outcome was observed in the standard tedizolid dose group ^135^.

***Telavancin*.** Telavancin is a semisynthetic lipoglycopeptide derivative of vancomycin with dose-dependent bactericidal activity against *enterococ*ci. It inhibits peptidoglycan synthesis by binding to D-alanyl-D-alanine and disrupts the cell membrane, increasing its permeability ^136^. Telavancin retains activity against GP organisms with decreased susceptibility to vancomycin. It is active against vancomycin-susceptible *Enterococcus faecalis* and *E. faecium* (MIC90 ≤1 µg/mL) ^137^, but, when tested against VRE strains, telavancin MICs were significantly elevated (MIC90 8–16 µg/mL) ^138^. Telavancin showed more potent activity against *vanB* strains (MIC ≤2 µg/mL) than against *vanA* strains (MIC ≤16 µg/mL) ^139^. Telavancin has a relatively prolonged half-life of 6.9–9.1 hours that allows for daily dosing, usually of 10 mg/kg/day. Dosage adjustments (7.5 mg/kg/day) are required for patients with creatinine clearance <50 mL/minute. Based on its comparable efficacy to vancomycin for the treatment of complicated skin and skin structure infection (SSTI) and pneumonia caused by GP cocci, telavancin received FDA approval for these indications in 2009 and 2013, respectively ^140, 141^. However, adverse events, including nephrotoxicity, which is comparable to that of vancomycin, have limited its use. No clinical data are available addressing the use of telavancin in invasive VRE infections and bacteremia. Finally, since most vancomycin-resistant *E. faecium* isolates possess the *vanA* gene, its role in the treatment of invasive VRE disease is limited.

***Dalbavancin*.** As a lipoglycopeptide, dalbavancin possesses a long lipophilic side chain that inserts into the bacterial cell membrane, enhances its affinity to the target site, and markedly prolongs its half-life. Its dual mechanism of action increases its *in vitro* activity against *enterococci* ^142^. In an evaluation of nearly 82,000 GP isolates, dalbavancin was over 16-fold more potent than vancomycin. MIC90 values against vancomycin-sensitive *E. faecalis* and *E. faecium* were of 0.06 and 0.12 mg/L, respectively. However, similar to telavancin, dalbavancin does not bind peptidoglycan precursors ending in D-Ala-D-Lac and only has significant activity against VRE isolates with the uncommonly encountered (in the US) *vanB* phenotype ^143, 144^, thus limiting its usefulness.

Dalbavancin was approved by the US FDA in 2013 for the treatment of ABSSSI ^145^. Its 181-hour half-life allows for weekly administration; it has a dual route of elimination demanding dose adjustments in renal dysfunction but not in hepatic failure. When evaluated in clinical trials for the treatment of SSTI, dalbavancin once weekly demonstrated non-inferiority with similar rates of both safety and efficacy compared with linezolid twice daily ^145– 147^. Its efficacy compared to that of vancomycin was evaluated in 75 patients with catheter-related BSIs caused by GP bacteria ^148^. Although overall efficacy was higher with dalbavancin (87% versus 50%), *enterococci* bacteremia was underrepresented in the study, with only two patients in the dalbavancin arm and three in the vancomycin arm, and organism-specific efficacy was not mentioned. Moreover, the role of dalbavancin, as with telavancin, is limited by its relatively lesser activity against *vanA*-carrying strains.

***Oritavancin*.** Oritavancin, approved by the FDA for ABSSSI in 2014, appears to be more promising for VRE infections. This antibiotic is a synthetic derivative of the natural glycopeptide chloroeremomycin. Its structural additions allow improved binding to the peptidoglycan precursor D-Ala-D-Ala but also to D-Ala-D-Lac necessary for the inhibition of cell wall synthesis and yielding significant activity against VRE carrying either *vanB* or *vanA* ^149, 150^. This improved binding to the cell wall assembly apparatus, coupled with its membrane effects as the result of binding of its lipophilic side chain, mediates potent bactericidal activity against both growing cells and biofilms.

Morrissey and colleagues collected 866 GP bacterial isolates from several countries in western Europe to evaluate their susceptibilities to oritavancin compared to other commercially available agents. Oritavancin was capable of inhibiting all isolates, including 101 VRE, at concentrations of 0.25 mg/L or less, confirming the potent *in vitro* activity of the drug even against GP bacteria resistant to newer agents like linezolid and daptomycin ^151^.

A terminal half-life of 393 hours, together with its post-antibiotic effect, allows single-dose administration of this drug for many infections ^152^. Although dose adjustments are not needed in mild-to-moderate renal and hepatic impairment, dosing in severe hepatic and renal dysfunction has not been studied. In the initial clinical trial evaluating the safety and efficacy of oritavancin for the treatment of ABSSSI, patients received oritavancin 1,200 mg as a single dose or vancomycin twice daily for 7–10 days. Both safety and efficacy end points, including reduction of lesion size, were comparable in both arms. Compared to a dose of 12 mg/kg/day of daptomycin, a single 1,200 mg dose of oritavancin demonstrated less rapid but more sustained bactericidal activity against vancomycin-resistant *E. faecium* isolates after 72 hours in an *in vitro* PK/pharmacodynamic (PD) model ^153^. However, there are no clinical data available addressing the use of oritavancin in VRE invasive infections, including VREB, and the optimal dosing regimen for these indications is unknown ^151^. Finally, the degree and mechanisms of resistance to oritavancin are not fully characterized. Thus, although promising, the role of oritavancin in the treatment of VRE infections remains to be established.

***Tigecycline*.** Tigecycline is a minocycline-derived glycylcycline with an N-alkyl-glycylamido group substitution, which allows it to have activity against tetracycline-resistant GN and GP organisms, including VRE ^154^. Tigecycline is highly active against *enterococci in vitro*. In a recent surveillance study ^155^, all *Enterococcus* species were sensitive to tigecycline with an MIC90 of 0.25 µg/mL, which is the breakpoint for *E. faecalis* (also known as VSE) established by the FDA and European Committee on Antimicrobial Susceptibility Testing ^156, 157^. Susceptibility breakpoints have not been set for other *Enterococcus* species such as *E. faecium*.

Tigecycline, which is bacteriostatic, has a large volume of distribution (range: 7–17 L/kg), leading to high concentrations in tissue but low concentrations in serum ^154^. These characteristics partially explain why it is not indicated for the treatment of VRE infections and should not be given in monotherapy for VREB ^158, 159^. In addition, tigecycline has carried a black box warning since 2013 for increased all-cause mortality, observed during an FDA meta-analysis evaluating tigecycline across all indications, noted in patients treated for ventilator-associated pneumonia ^157^. In small clinical trials evaluating tigecycline for complicated intra-abdominal infections and SSTI, it demonstrated similar efficacy relative to comparators (imipenem/cilastatin and vancomycin with aztreonam for each indication, respectively) in patients with concomitant bacteremia; however, no cases of VREB were reported in these studies ^160, 161^. Although tigecycline may be useful in patients with difficult-to-treat infections who have no superior treatment alternatives, it is lacking in clinical data to support its use for VRE infections, especially for VREB.

### Antibiotic combinations ^162^

***Daptomycin plus β-lactams*.** The synergy of several β-lactam antibiotics in combination with daptomycin against VRE has been evaluated *in vitro*. The mechanism by which this occurs is similar to that described in cases of methicillin-resistant *Staphylococcus aureus* ^162, 163^, although resistance mechanisms for *enterococci* are more complex. In the presence of β-lactams, the charge of the bacterial surface becomes more negative, which facilitates binding of the daptomycin-calcium complex, even in cases of non-susceptibility to daptomycin. This leads to enhanced membrane depolarization, increased fluidity, and susceptibility to killing by cationic calcium-daptomycin and a diverse range of human cationic antimicrobial peptides, notably cathelicidin LL-37 ^164, 165^. Synergistic effects have been observed *in vitro* with ampicillin, ceftriaxone, ceftobiprole, and ceftaroline, even in the presence of resistance to these β-lactams ^164– 167^. However, when combinations with ampicillin, ceftriaxone, or ceftaroline have been tested against DNS vancomycin-resistant *E. faecium* strains, it is unclear which β-lactam is preferred. Both ampicillin and ceftaroline have demonstrated superior synergism in separate studies from the same group ^164– 168^. More recent studies have shown that, compared to other β-lactams, ertapenem may have more synergistic activity, especially for DNS VRE strains ^169, 170^.

Based on these data, combination treatment has been explored with anecdotal reports of success. Sakoulas *et al*. reported a case of a hemodialysis patient with ampicillin-resistant vancomycin-resistant *E. faecium* infective endocarditis failing seven days of therapy with daptomycin plus linezolid despite susceptibility to each ^164^. Treatment with high-dose daptomycin (12 mg/kg) plus ampicillin 1,000 mg every six hours resulted in blood culture clearance within 24 hours. The authors also noted that, with ampicillin, daptomycin MICs decreased from 1.0 to 0.38 mg/L. This combination was also successful in an 89-year-old female treated for six weeks for *E. faecalis* infective endocarditis susceptible to both ampicillin and daptomycin ^171^. Combined with ceftaroline at a dose of 600 mg every eight hours, daptomycin (at 8 mg/kg dosing) was successfully used to treat a 63-year-old man with E. faecalis endocarditis unresponsive to other therapies ^172^. In contrast, the combination of ceftriaxone and daptomycin failed in a case of *E. faecalis* endocarditis ^173^.

***Daptomycin plus fosfomycin*.** Several studies have examined the synergy between daptomycin combined with intravenous fosfomycin. In two time-kill assays, the combination displayed bactericidal activity against all strains of VRE with greater killing effect than monotherapy ^174^ and was more potent than combinations of daptomycin with ampicillin or linezolid ^175^. In 72-hour *in vitro* PK/PD models, the addition of fosfomycin to both 8 and 12 mg/kg/day of daptomycin resulted in significantly greater bactericidal activity than daptomycin alone against all daptomycin-susceptible (DS) isolates and prevented the development of daptomycin resistance in two out of three of these isolates. However, the higher daptomycin dose of 12 mg/kg/day was necessary to maintain bactericidal activity for the entire 72 hours. No synergy was observed against DNS strains ^176^. The only *in vivo* data available on this combination come from rat models of vancomycin-resistant *E. faecalis* endocarditis with high-level gentamicin resistance, in which sterilization of valves was achieved more frequently with the combination therapy ( *P* = 0.3) ^177^. However, it is worth noting that an intravenous formulation of fosfomycin is not available in the US, although it is currently in late-stage clinical trials ( Table 2).

**Table 2.** Key points.

• Vancomycin-resistant enterococci (VRE) colonization is, at many centers, common and increasing in frequency. • VRE colonization surveillance is not a standard practice at all centers but is recommended in the presence of ongoing strain transmission. • Patients colonized with VRE, especially with its dominance in the intestinal microbiome, have a high rate of development of VRE bacteremia (VREB), and this is most likely to occur during receipt of broad-spectrum antibiotics. • VREB is associated with excess mortality, but attributable mortality appears to be limited, indicating that it may be a surrogate marker of mortality, which is more related to the presence of comorbidities. • While the presence of colonization is a predictor of risk of VREB, there is no evidence that therapy directed at VRE in patients with persisting fever and negative cultures improves outcomes. • Daptomycin and linezolid are the current mainstays of therapy for VREB; emergence of isolates with reduced susceptibility to both agents may pose future challenges to treatment • In patients with persisting bacteremia despite appropriate monotherapy, a combination of daptomycin with a β-lactam antibiotic, such as ampicillin, can be considered.

***Daptomycin plus tigecycline*.** Although monotherapy with tigecycline is not recommended, its combination with daptomycin has been associated with favorable outcomes in several case reports ^178– 181^. The first case report was of a 62-year-old male with infective endocarditis due to *E. faecium* resistant to ampicillin, chloramphenicol, linezolid, vancomycin, and quinupristin/dalfopristin and lacked both gentamicin and streptomycin synergy ^178^. Sterilization of the bloodstream was achieved within three days of starting daptomycin and tigecycline. Similarly, a 39-year-old female with VRE endocarditis— *E. faecium* resistant to linezolid and with a daptomycin MIC of 4 mcg/mL—failed treatment with daptomycin at 8 mg/kg/day dosing ^179^. Microbiological cure was achieved with the addition of tigecycline, and blood cultures remained negative nine weeks after discharge. Jaspan *et al*. reported a case of a 21-month-old female with refractory acute biphenotypic leukemia who developed VREB ^180^. She was treated with linezolid plus daptomycin with resultant bloodstream sterilization but continued to be febrile and eventually her cerebrospinal fluid (CSF) grew a linezolid-resistant VRE strain. Treatment with intravenous and intraventricular daptomycin plus tigecycline resulted in sterilization of the CSF after 48 hours.

***Linezolid combinations*.** Gentamicin, rifampin, and doxycycline have displayed potential benefit when used with linezolid, but evidence supporting linezolid combinations is sporadic and contradictory ^182– 185^.

## Conclusions

VRE has become a major cause of bacteremia in HSCT recipients. Although VRE infection does not significantly increase mortality in this population, it has important implications in HSCT outcomes. Colonization, as a precondition to VRE invasive disease, can be used to identify patients at high risk of bacteremia. Further studies are needed to examine the utility and impact of routine stool screening—especially with PCR—and isolation of colonized patients. How best to utilize data obtained from surveillance cultures remains a controversy in clinical practice. In centers with high rates of colonization and progression to VREB, empiric anti-VRE therapy may be warranted when antibiotics against GP bacteria are necessary. Further investigation is warranted to establish a more precise algorithm for indications of empirical VRE-active therapy in febrile HSCT recipients, incorporating factors such as colonization status, antimicrobial exposures, and patient/treatment factors. Targeted therapy against VRE continues to be centered in the use of daptomycin and linezolid. Despite the advent of new agents with excellent *in vitro* activity against VRE and theoretical benefit of antibiotic combinations, clinical data supporting their use for invasive VRE infections are still lacking. In patients with persisting bacteremia despite appropriate monotherapy, a combination of daptomycin with a β-lactam antibiotic, such as ampicillin, can be considered.

## Search criteria

"VRE" [tw] OR "vancomycin resistant enterococcus" [tw] AND "stem cell" [tw] - 56

"VRE"[tw] OR "vancomycin resistant enterococcus"[tw] AND ("stem cell"[tw] OR "bone marrow*"[tw]) – 84
